# Effect of diabetes self-management education on glycaemic control among type 2 diabetic patients at a family medicine clinic in Kenya: A randomised controlled trial

**DOI:** 10.4102/phcfm.v10i1.1762

**Published:** 2018-11-19

**Authors:** Catherine W. Gathu, Jacob Shabani, Nancy Kunyiha, Riaz Ratansi

**Affiliations:** 1 Department of Family Medicine, The Aga Khan University, Kenya; 2 Department of Medicine, The Aga Khan University, Kenya; 3 Department of Family Medicine, The Aga Khan University, Dar es Salaam, Tanzania

## Abstract

**Background:**

Diabetes self-management education (DSME) is a key component of diabetes care aimed at delaying complications. Unlike usual care, DSME is a more structured educational approach provided by trained, certified diabetes educators (CDE). In Kenya, many diabetic patients are yet to receive this integral component of care. At the family medicine clinic of the Aga Khan University Hospital (AKUH), Nairobi, the case is no different; most patients lack education by CDE.

**Aim:**

This study sought to assess effects of DSME in comparison to usual diabetes care by family physicians.

**Setting:**

Family Medicine Clinic, AKUH, Nairobi.

**Methods:**

Non-blinded randomised clinical trial among sub-optimally controlled (glycated haemoglobin (HbA1c) ≥ 8%) type 2 diabetes patients. The intervention was DSME by CDE plus usual care versus usual care from family physicians. Primary outcome was mean difference in HbA1c after six months of follow-up. Secondary outcomes included blood pressure and body mass index.

**Results:**

A total of 220 diabetes patients were screened out of which 140 met the eligibility criteria and were randomised. Around 96 patients (69%) completed the study; 55 (79%) in the DSME group and 41 (59%) in the usual care group. The baseline mean age and HbA1c of all patients were 48.8 (standard deviation [SD]: 9.8) years and 9.9% (SD: 1.76%), respectively. After a 6-month follow-up, no significant difference was noted in the primary outcome (HbA1c) between the two groups, with a mean difference of 0.37 (95% confidence interval: -0.45 to 1.19; *p* = 0.37). DSME also made no remarkable change in any of the secondary outcome measures.

**Conclusion:**

From this study, short-term biomedical benefits of a structured educational approach seemed to be limited. This suggested that offering a short, intensified education programme might have limited additional benefit above and beyond the family physicians’ comprehensive approach in managing chronic conditions like diabetes.

## Introduction

Diabetes self-management education (DSME) is a key component of diabetes care.^1^ It offers good glycaemic and metabolic control, which is essential for preventing long-term complications such as retinopathy, nephropathy, neuropathy and cardiovascular diseases, and premature mortality.^2^ It should be offered right from diagnosis,^3^ especially in low-resource settings where it is known to have positive effects on knowledge of diabetes, glycaemic control and behavioural outcomes.^4^

Diabetes self-management education complements diabetes medication, which has failed to control blood glucose despite its accessibility and proven efficacy in many type 2 diabetic patients (HBA1c ≤ 6.5%). An estimated 80% of type 2 diabetes patients in Kenya^5^ and Cape Town, South Africa, respectively^6^ have poor glycaemic control contributing to an increase in diabetes complications. This increase necessitates effective diabetes prevention and control measures including DSME, especially noting that a 1-point improvement of HbA1c is associated with a 20% and 30% – 40% decrease in macro-vascular and micro-vascular complications, respectively.^7^ Moreover, the knowledge deficit regarding diabetes is alarming. In Kenya, 71% of diabetic patients have poor knowledge of diabetes^8^ while two-thirds are undiagnosed and living in the community completely unaware of their illness, indicating a huge knowledge deficit even among affected individuals.^9^

In many sub-Saharan countries, there is a paucity of information regarding educational interventions for the prevention of diabetes complications, hence, a majority of diabetic patients are yet to receive this integral component of care, thereby putting them at risk of diabetes-associated complications.^10^ Unstructured DSME is mainly offered on an ad hoc basis in the hospital’s waiting area as opposed to a well-structured educational programme^11^ because of few trained and accessible certified diabetic educators, financial constraints and lack of awareness among patients and health professional about the need for DSME.^11^ Thus, the objective of the study was to evaluate whether a structured DSME in addition to usual care improved glycaemic control as compared to usual care only among sub-optimally controlled type 2 diabetes patients.

## Methods

### Study design and setting

This was a non-blinded, randomised controlled clinical trial, carried out between April 2015 and September 2015, involving sub-optimally controlled type 2 diabetes patients attending the family medicine clinic (FMC) at the Aga Khan University Hospital (AKUH), Nairobi. The FMC is a private, urban-based, primary care clinic, located within a tertiary, teaching and referral hospital in Nairobi serving a multi-ethnic population of mostly middle and high socio-economic status patients.

### Study population

We recruited and screened patients from FMC diabetes registry, who had sub-optimally controlled type 2 diabetes, defined as HbA1c ≥ 8% and were aged 18–65 years. We excluded patients with other types of diabetes, diabetes-related complications and anaemia at last haemoglobin count since these would confound the results. Diabetes-related complications were screened based on the last laboratory check-up of their kidney function test and estimated glomerular filtration rate calculation using Modification in Diet in Renal Disease (MDRD) study equation, the last eye check-up and the clinical notes from their family physician excluding any neuropathic symptoms or symptoms of other complications. Anaemia was excluded because it affects HbA1c levels; with anaemic patients having short-lived red blood cells, so those who are diabetic, will test with falsely low HbA1c levels.

### Study interventions

#### Usual care

Usual care by the family physician was delivered as per the usual consultation practise at the FMC with no modification. It entailed a 20–30 min standard doctors’ consultation where the recent HbA1c level and medication compliance were reviewed, and a brief informal patient-tailored diabetes education was offered. This enabled the individual an opportunity to learn about self-management in a flexible and informal way. There was no structure to it and the information was offered according to what the patient requested to know as well as what the doctor thought would have been important for the patient to know, during that consultation. Print, audio-visual and online patient education materials were used depending on the provider. Three family physicians who are routinely involved in diabetes management at the FMC were included in this study and usual care was not standardised among them. Subsequently, follow-up with their family physician was arranged on a quarterly basis.

#### Diabetes self-management education

The intervention group received the usual consultation from their family physician and referral to a certified diabetes educator, for individualised structured DSME training. An empowerment and interactive teaching model was used with focus on behavioural assessment, goal-setting and problem-solving to promote autonomous self-regulation for better health and quality of life. The empowerment-based diabetes education programme is tailored to include strategies that are evidence-based, culturally appropriate and integrated, with emphasis on patient-centredness.^12^ This intervention can be conducted across different educational and clinical settings to address the unique challenges of each diabetic patient. A standard AKUH clinical sheet was used while delivering the education (Appendix 2) to ensure all core topics were covered.

For this study, two certified diabetes educators with level four designation offered the individualised DSME sessions.^13^ The education content included the American Association of Diabetes Educators (AADE) 7-core self-care behaviours; being active, nutrition, monitoring blood glucose and adherence to medication, among other topics. The participants were scheduled to attend three one-hour sessions after every six weeks. The first session was arranged within a month from their initial consultation with their primary family physicians. At the end of the sessions, the participants received a patient guide to diabetes booklet and graphic material illustrating several self-care activities such as foot care. Subsequent consultations were mainly feedback sessions, aimed at reviewing previously discussed matters, reinforcing key messages, addressing challenges and providing additional information.

The patients also received telephone reminders, a week prior to their scheduled appointment with the diabetes educators, to ensure timely communication and confirmation of their visit. A hotline number was also availed to them to consult with the diabetic educator at any given time of the day. Follow-up with their primary care physician continued as usual, after every three months.

### Randomisation

A research assistant invited all the eligible patients for participation, and randomised them equally into two groups based on computer-generated random numbers and informed them of the assigned group. The randomisation allocation sequence remained concealed from the principal investigator and family physicians to further eliminate conscious or unconscious selection bias. After recruitment, the patients completed their demographic and medical history information on a standard data collection form (Appendix 1).

### Study outcomes

The primary outcome of the study was HbA1c, a form of haemoglobin that is measured primarily to identify the three-month average plasma glucose concentration as an indicator of diabetes control.^14^ HbA1c was tested at an accredited laboratory at the hospital on the family physician’s request on recruitment and after six months. The results of the test were retrieved from the lab records and tabulated in the standard data collection form. Before each of the HbA1c test, a research-trained nurse measured and documented the blood pressure, height and weight.

The blood pressure was measured using a calibrated digital sphygmomanometer. The nurse ensured that the patient was properly prepared and positioned prior to taking the reading; did not drink a caffeinated beverage or smoked 30 min before the test, sat quietly for 5 min before the test began and sat in a chair with feet on the floor and arms supported so that the elbow is at about heart level.

Height was measured, without shoes, to the nearest 0.1 cm using calibrated stadiometers. A sliding horizontal headpiece that adjusts to rest gently on the top of the head was used to obtain the measurement while the patient stood erect, with back on the wall, looking straight ahead. Weight was measured, without shoes and heavy outer garments, to the nearest 0.1 kg, using a calibrated weighing scale.

The secondary outcomes of the study included systolic and diastolic blood pressure, and body mass index (BMI), which was calculated as a person’s weight in kilograms divided by their height in metres squared at recruitment and after six months.

Reliability of the measurements was ensured by training the research nurses, involving only the trained research nurses in taking the measurements, ascertaining that measurements were taken accurately by the primary investigator through regular supervision and regular servicing and calibrating equipment as per the hospital guidelines.

### Sample size and statistical analysis

We estimated that 70 participants were needed in each group to have 80% capability to detect an absolute difference in HbA1c levels of 1% between groups at the 95% significance level, assuming a standard deviation (SD) of 2% and adjusting for an anticipated 10% dropout rate.

The baseline characteristics of the patients in the two groups were described using frequency distribution and descriptive statistics and compared using the student’s *t*-test for continuous variables and chi-square test for categorical variables. The mean change after six months for all outcomes, was calculated in both groups and the significance tested using the student’s *t*-test for the mean difference between the intervention and control arm. All the analyses were performed using STATA (version 12.0).

### Ethical consideration

The Aga Khan University Hospital Nairobi Health Research Ethics Committee approved the study (ethical clearance number: 2013/REC-49[v6]) and all participants provided written informed consent.

## Results

A total of 220 patients from the family medicine clinic diabetes registry were screened for eligibility and 140 (64%) met the eligibility criteria and were randomised between April 2015 and September 2015. After a six-month follow-up, 96 patients (69%) had complete data that were used for final analysis (Figure 1). The loss to follow-up was significantly more common in the usual care group than the intervention group (41% vs. 21%; *p* = 0.005).

**FIGURE 1:**
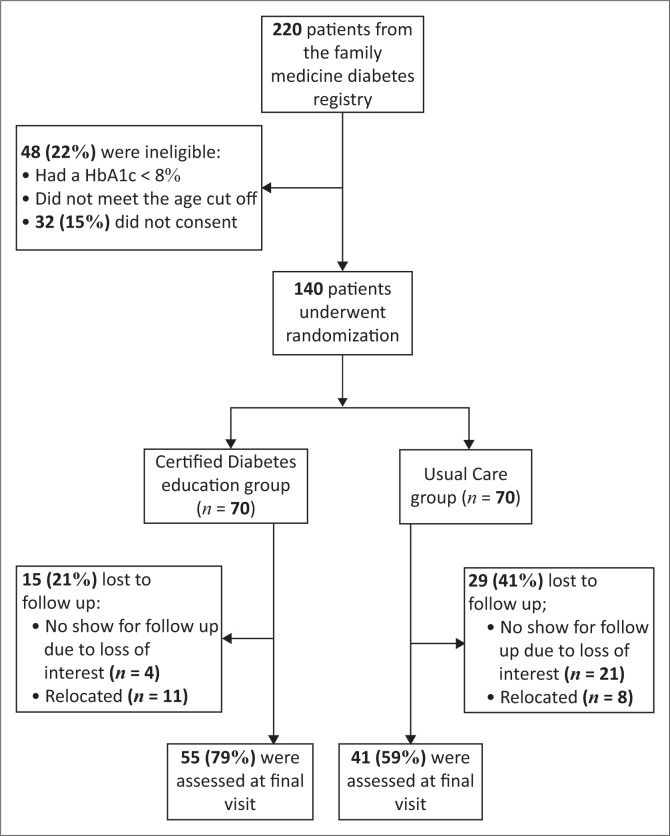
Participants flow in the study.

At recruitment, there were 78 (56.0%) males and 62 (44.0%) females, with a mean age of 48 years (SD: 9.8 years). The baseline mean HbA1c was 9.8% in the intervention group compared to 10.0% in the usual care group. Body mass index was 28.5 kg/m^2^ versus 28.8 kg/m^2^ and blood pressure was 134 out of 80 mmHg versus 134 out of 82 mmHg in the intervention versus control group, respectively (Table 1). The baseline demographic and clinical characteristics were similar in both groups with the exception of the prevalence of hypertension (49% in intervention arm vs. 29% in control arm, *p* = 0.02). Those lost to follow-up were also more likely to be hypertensive compared to those who completed follow-up (*p* = 0.003) but did not differ with respect to other characteristics (Table 2).

**TABLE 1:** Socio-demographic and baseline characteristics of patients with type 2 diabetes mellitus participating in a randomised control trial of diabetes self-management education compared to usual care, overall and stratified by study arm.

Characteristics	Total (*N* = 140)				Intervention (*N* = 70)				Usual care (*N* = 70)				*p*
	Mean	SD	*n*	%	Mean	SD	*N*	%	Mean	SD	*n*	%	
**Mean age – years**	48.8	9.8	-	-	50.2	9.93	-	-	47.5	9.54	-	-	0.100†
**Gender**													
Male	-	-	78	55.7	-	-	41	59.0	-	-	37	53.0	0.490*
Female	-	-	62	44.3	-	-	29	41.0	-	-	33	47.0	
**Metabolic profile**													
BMI	28.7	3.76	-	-	28.5	3.73			28.8	3.8			0.620†
**Baseline blood pressure – mm Hg**													
Systolic	134.6	13.54	-	-	134.3	14.63	-	-	134.8	12.46			0.840†
Diastolic	81.7	10.85	-	-	80.7	10.53	-	-	82.6	11.16			0.300†
**Co-morbid**	-	-	-	-	-	-	-	-	-	-			
Hypertension	-	-	54	39.0	-	-	34	49.0	-	-	20	29.0	0.020*
**Baseline HbA1c**	9.9	1.76	-	-	9.7	1.78	-	-	10	1.74	-	-	0.230†
**Duration of diabetes**													
< 5 years	-	-	69	49.3	-	-	34	49.0	-	-	35	50.0	0.620*
5–10 years	-	-	31	22.1	-	-	14	20.0	-	-	17	24.0	
>10 years	-	-	40	28.6	-	-	22	31.0	-	-	18	26.0	
**Mode of medication**													
Diet and exercise	-	-	79	56.4	-	-	11	16.0	-	-	17	24.0	0.200*
Oral	-	-	3	2.1	-	-	42	60.0	-	-	37	53.0	
Oral and Insulin	-	-	29	20.7	-	-	14	20.0	-	-	15	22.0	
Insulin	-	-	29	20.7	-	-	3	4.0	-	-	1	1.0	
**Level of education**													
Primary	-	-	6	4.3	-	-	6	9.0	-	-	1	1.0	0.160*
Secondary	-	-	27	19.3	-	-	12	17.0	-	-	15	21.0	
Tertiary	-	-	107	76.4	-	-	51	74.0	-	-	54	78.0	
**Currently smoking**	-	-	9	6.4	-	-	5	7.0	-	-	4	6.0	0.730*
**Consuming alcohol**	-	-	54	38.6	-	-	25	36.0	-	-	29	41.0	0.490*
**Lost to follow-up**	-	-	44	31.0	-	-	15	21.0	-	-	29	41.0	0.005

BMI, body mass index; SD, standard deviation; HbA1c, glycated haemoglobin; *N*, sample number.

* , chi-square.

† , Student’s *t*-test.

**TABLE 2:** Characteristics of patients who returned for follow-up at six months versus those lost to follow-up.

Characteristics	Baseline								*P*
	Returned for follow-up (*N* = 96)				Lost to follow-up (*N* = 44)				
	Mean	SD	*n*	%	Mean	SD	*n*	%
**Mean age - years**	49.2	9.59	-	-	47.9	10.29	-	-	0.460†
**Gender**									
Male	-	-	55	37.0	-	-	23	52.0	0.580*
Female	-	-	41	43.0	-	-	21	48.0	
**Metabolic profile**									
BMI	28.7	4.20	-	-	28.5	2.56	-	-	0.730†
**Baseline blood pressure - mmHg**									
Systolic	134.2	14.95	-	-	135.3	9.92	-	-	0.670†
Diastolic	81.9	10.25	-	-	80.9	12.17	-	-	0.610†
**Co-morbid**									
Hypertension	-	-	45	47.0	-	-	9	20.0	0.003*
**Baseline HbA1c - Duration of diabetes**	9.8	1.72	-	-	10	1.88	-	-	0.660†
< 5 years	-	-	46	48.0	-	-	23	52.0	0.300*
5–10 years	-	-	19	20.0	-	-	12	27.0	
>10 years	-	-	31	32.0	-	-	9	21.0	
**Mode of medication**									
Diet and exercise	-	-	14	15.0	-	-	14	32.0	0.070*
Oral	-	-	58	60.0	-	-	21	48.0	
Oral and Insulin	-	-	21	22.0	-	-	8	18.0	
Insulin	-	-	3	3.0	-	-	1	2.0	
**Level of education**									
Primary	-	-	6	5.0	-	-	2	2.0	0.330*
Secondary	-	-	14	15.0	-	-	13	30.0	
Tertiary	-	-	76	80.0	-	-	29	68.0	
**Currently smoking**	-	-	7	7.0	-	-	2	5.0	0.540*
**Consuming alcohol**	-	-	33	34.0	-	-	21	48.0	0.130*

BMI, body mass index; SD, standard deviation; HbA1c, glycated haemoglobin; *N*, sample number.

* Chi-square.

† , Student’s *t*-test.

After six months, the mean HbA1c had decreased significantly in both arms, from 9.8% to 8.8% in the intervention arm (mean difference: -0.98, SD: 2.29) and 9.9% to 9.3% in the control arm (mean difference: -0.60, SD: 1.59). However, the magnitude of this decrease did not differ between the two arms (*p* = 0.37) (Table 3). Blood pressure and BMI did not change from baseline to 6 months of follow-up, and the mean difference between baseline and follow-up in these outcomes did not differ significantly between study arms (Table 3).

**TABLE 3:** Summary of the mean differences of primary and secondary outcomes between both groups.

Outcome	Control group (*n* = 41)						Intervention group (*n* = 55)						Difference in the change between baseline and 6 months, intervention – control group		*p*
	Baseline		6 months		Mean difference between baseline and 6 months		Baseline		6 months		Mean difference				
	Mean	SD	Mean	SD	Mean	SD	Mean	SD	Mean	SD	Mean	SD	Mean	SD
HbA1c (%)	9.9	1.45	9.3	1.75	−0.6	1.54	9.8	1.9	8.8	1.89	−0.98	2.29	0.37	0.41	0.37
SBP (mmHg)	134.1	13.6	133.8	11.54	−0.29	11.16	134.3	15.9	132.6	15.32	−1.78	13.47	1.49	2.59	0.57
DBP (mmHg)	83.5	10.07	82.6	9.86	−0.9	11.48	80.8	10.32	78	9.04	−2.80	10.37	1.89	2.24	0.39
BMI	28.9	4.48	29.3	4.55	0.41	0.76	28.6	4.03	28.9	3.87	0.37	1.21	0.04	0.22	0.86

HbA1c, glycated haemoglobin; CI, confidence interval; BMI, body mass index; SBP, systolic blood pressure; DBP, diastolic blood pressure.

## Discussion

In this study, six months of individualised DSME did not significantly improve the glycaemic and metabolic control of the sub-optimally controlled type 2 diabetes patients. Similar findings were observed in a review of six studies comparing individual education to usual care over a 12–18-month period.^15^ However, a significant improvement in glycaemic control was noted in a subgroup analysis of three studies involving participants with a higher mean baseline HbA1c greater than 8%.^15^ The lack of similar results in our study population of patients with HbA1c ≥ 8% may be due to heterogeneity between trials in method of delivery, intervention duration as well as the content of the education offered in the self-management education programmes.

The DESMOND trial, another large multicentre randomised control clinical trial also found no difference in HbA1c, up to 12 months after diagnosis.^16^ This goes on to support our study findings. In regard to frequency of sessions, this trial only delivered the educational content during the first two days of the study, for 6 h. Our study entailed a continuous approach to the provision of the educational content across the six-month period, with 3 h of contact time distributed longitudinally. Both methods of delivery did not seem to favour the intervention. Two systematic reviews which included more than a hundred randomised control trials in each review found significant HbA1c improvement in studies with a contact time of 10 h or longer.^17,18^ Contact hours of educational content provision as well as frequency are plausible determinants of effectiveness of a DSME programmes.^19^ This has not been standardised, hence is a key challenge in the provision and assessment of such programmes and needs to be addressed.

Self-management education should be person-centred and population-specific.^20^ Culture-specific needs assessment ought to be carried out prior to implementing a DSME programme, otherwise, the current approach to DSME will not meet the patients’ expectations.^21^ This study recorded a high dropout rate of 31% with similar reasons in both groups. Loss of interest in diabetes education was cited by most of the patients who were lost to follow-up. If patients feel that their needs are not accounted for during the education sessions, or the content is not useful to them, they are likely to be less interested and subsequently fail to show up in future. This may explain why many patients dropped out of the study and reinstate the need to do a needs assessment prior to designing DSME programmes and incorporate the patients’ expectations, to enhance cultural relevance.

A South African study explored the effectiveness of a group educational programme.^22^ Similarly, no significant effect on primary outcomes including HbA1c, was noted after a one-year follow-up period. Diversity in the provision of the diabetes education (group vs. individualised) exists and it is not clear even within the African context, whether one modality is superior to the other. Future studies should investigate this aspect. This study was also limited by high dropout rates and strategies to maximise the uptake of such programmes in sub-Saharan Africa needs to be explored.

This study had some important limitations that may have contributed to the negative outcome. First, the study was entirely carried out in one setting, with a significant risk of ‘cross-contamination’ between control and intervention groups. Second, the follow-up period was only 6 months which is shorter than most of the similar studies which typically have a 1–3-year follow-up. Third, most of our study participants had very long-standing diabetes that may contribute to biomedical outcomes, diabetes education notwithstanding.

Fourth, there was no evaluation of the DSME intervention provided by the diabetes educators. The educators were not supervised during any of the education sessions to assess fidelity to the planned educational programme. Fifth, this study did not address qualitative issues surrounding effectiveness of an educational approach, such as prior knowledge, knowledge acquired, improvements in self-care activities, psychological factors, among others, which would have been important to further enrich this discussion. Lastly, potential confounders to the learning process include motivation and attitude, emotional barriers especially for newly diagnosed patients, family and social support, all of which were also not factored in assessing the effectiveness of the intervention.

## Conclusion

Although individualised diabetes education has been shown to improve outcomes in some studies, variability in duration and frequency of the intervention, content and the method of delivery exist, and are important determinants to the effectiveness of a programme.^20^ In this study, DSME did not show statistically significant improvement in glycaemic and metabolic control.

Diabetes being a chronic condition, self-management education needs to be an ongoing, lifelong process of facilitating the knowledge and skills necessary for diabetes self-care. From this study, short-term biomedical benefits of a structured educational approach seem to be limited.

This suggests that offering a short, intensified education programme may have limited additional benefit above and beyond the family physicians comprehensive approach in managing chronic conditions like diabetes. However, robust, quality-assured DSME programmes would need to be in place to further strengthen this observation. Diabetes education curriculums need to be developed within the African context taking into account the unique challenges for this population and exploring factors to increase uptake.

## Figures and Tables

**FIGURE 1-A2:**
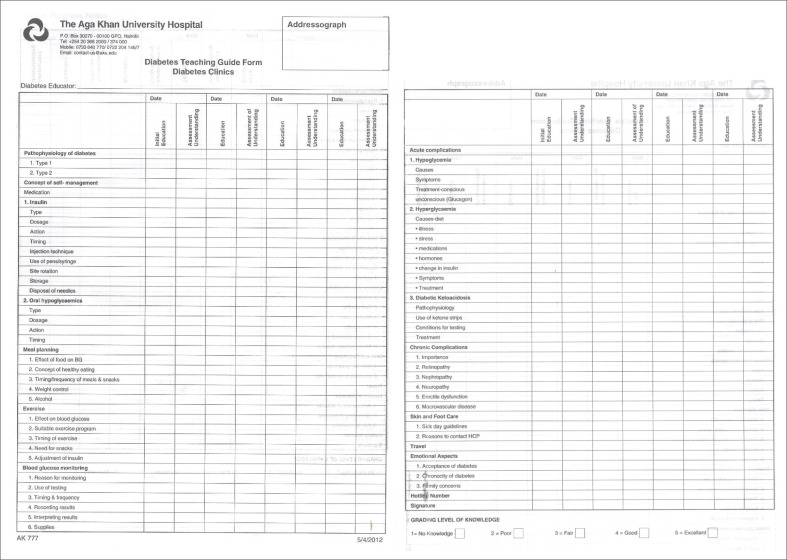
Diabetes education guide.
